# *CXCR2* May Serve as a Useful Index of Disease Activity in Interstitial Lung Disease Associated With Primary Sjögren’s Syndrome

**DOI:** 10.3389/fmolb.2021.640779

**Published:** 2021-05-13

**Authors:** Xiaofang Zhu, Saisai Lu, Lixia Zhu, Mengjiao Yu, Tingting Wei, Xiaochun Zhu, Dan Chen, Chengshui Chen

**Affiliations:** ^1^ Department of Rheumatology, The First Affiliated Hospital of Wenzhou Medical University, Wenzhou, China; ^2^ Department of Pneumology, The First Affiliated Hospital of Wenzhou Medical University, Wenzhou, China

**Keywords:** primary Sjӧgren’s syndrome, interstitial lung disease, CXCR2, ESSDAI, DLCO

## Abstract

**Background:** Primary Sjögren’s syndrome (pSS) is a chronic systemic autoimmune disease characterized by typical autoantibody production and lymphocytic-mediated exocrine gland damage. Interstitial lung disease (ILD) is a common complication of pSS and can be associated with a poor prognosis. However, the pathogenesis of ILD in pSS is still unclear.

**Methods:** In this study, we used RNA sequencing to investigate the gene-expression profile of the minor salivary glands (MSGs) from 36 patients with ILD-pSS and 128 patients with non-ILD-pSS.

**Results:** In the remarkably enriched chemokine-mediated signaling pathway, C-X-C motif chemokine receptor 2 (*CXCR2*), a receptor for interleukin-8, which participates in the activation of neutrophils, was found to be significantly elevated in both MSG and plasma from pSS patients with vs. without ILD (*p* < 0.001). Furthermore, the *CXCR2* expression level in MSG and plasma was significantly associated with the diffusing capacity of the lungs for carbon monoxide, erythrocyte sedimentation rate, and EULAR Sjögren’s Syndrome disease Activity Index in ILD-pSS.

**Conclusion:** Therefore, with its potential role in ILD progression in patients with pSS and its strong association with clinical manifestations of the disease, *CXCR2* may serve as a useful index for disease activity in ILD associated with pSS.

## Introduction

Primary Sjögren’s syndrome (pSS) is a systemic autoimmune disease characterized by the presence of organ-specific or non-specific autoantibodies and lymphocyte-mediated exocrine gland damage (Mavragani and Moutsopoulos, 2014). Although the major clinical manifestation of pSS is sicca symptoms, about 40% of patients develop a wide spectrum of extra-glandular involvement (Seror et al., 2014). Extra-glandular manifestations, especially interstitial lung disease (ILD), are associated with a poor prognosis and increased mortality in pSS (Roca et al., 2017; Sambataro et al., 2020). ILD has been observed in 9–75% of patients with pSS (Kelly et al., 1991; Bellido-Casado et al., 2011; Nannini et al., 2013; Palm et al., 2013; Kreider and Highland, 2014; Jin et al., 2019; Natalini et al., 2019). In addition, ILD has been reported to have a cumulative incidence of 10% at 1 year after diagnosis of pSS, 20% after 5 years, and 47% after 15 years of disease (Nannini et al., 2013). Therefore, there is an urgent need to develop new diagnostic markers as well as potential therapeutic targets to improve the outcomes of patients with pSS with ILD.

Minor salivary gland (MSG) biopsy is a cornerstone in the diagnosis of pSS (Daniels and Whitcher, 1994; Caporali et al., 2008; Vissink and Bootsma, 2016; Chiu et al., 2020). MSG biopsies exhibiting focal lymphocytic sialadenitis, based on a focal score of one or more lymphocytic foci (>50 lymphocytes/4 mm^2^), are considered to be of great significance for pSS (Daniels and Whitcher, 1994; Caporali et al., 2008). Furthermore, a focus score of ≥4 on an MSG biopsy is more frequently found in patients with ILD-pSS than in those with non-ILD-pSS (Kakugawa et al., 2018). Gene-expression profiling of MSGs has revealed a distinct gene-expression signature in patients with pSS compared with healthy control individuals (Hjelmervik et al., 2005). However, few studies have investigated differences in MSG gene expression between patients with ILD-pSS and those with non-ILD-pSS.

In this study, we investigated the clinical characteristics of and compared gene expression in MSGs from patients with ILD-pSS and non-ILD-pSS, with the aim of enhancing our understanding of the mechanisms underlying the progression of patients with pSS with ILD. In addition, we explored potential biomarkers for evaluating the disease severity of ILD-pSS in clinical practice.

## Materials and Methods

### Patients and Sample Preparation

Patients with ILD-pSS (*n* = 36) and non-ILD-pSS (*n* = 128) were recruited from the First Affiliated Hospital of Wenzhou Medical University, China, between 1 January 2018 and 30 June 2020. All patients fulfilled the 2016 American College of Rheumatology (ACR)/EULAR classification criteria (Shiboski et al., 2017) or 2012 ACR classification criteria (Shiboski et al., 2012) for pSS and were evaluated for systemic involvement. A diagnosis of ILD was based on the findings of high-resolution computed tomography scans of the chest (Dong et al., 2018), combined with medical history and physical examination. All patients also underwent a MSG biopsy for diagnostic purposes.

Salivary glands and plasma samples were both collected at the time of MSG biopsies. All samples, clinical data, and EULAR Sjögren’s Syndrome Disease Activity Index (ESSDAI) scores were obtained before the participants were given any systemic immunosuppressant or glucocorticoid to avoid confounding effects of these medications.

The study was approved by the ethics committee of the First Affiliated Hospital of Wenzhou Medical University (approval #16024), and all patients gave their written informed consent.

### RNA Extraction, cDNA Library Preparation, and Sequencing

Total RNA was isolated from frozen salivary gland samples using TRIzol Reagent (Invitrogen, Carlsbad, CA, United States) according to the manufacturer’s instructions. RNA purity was checked using a Nano Photometer spectrophotometer (IMPLEN, Westlake Village, CA, United States). The quality of the RNA used to construct the cDNA library was verified (RNA integrity numbers >9) using an RNA 6000 Nano kit with the Bioanalyzer 2100 system (Agilent Technologies, Santa Clara, CA, United States). With input material of 3 mg RNA per sample, we constructed sequencing libraries using the NEBNext Ultra RNA Library Prep Kit for Illumina (NEB, United States) and subsequently performed the sequence on an Illumina HiSeq platform. Then, using Cutadapt adapters and TrimGalore, raw reads were trimmed and low-quality reads were filtered. Quality-control reports of sequence reads were obtained using FastQC software. Finally, the sequencing data were aligned to the human reference genome “hg38” using STAR software, and the read count files were filtered with low expression and normalized using the DESeq2 package.

### Enzyme-Linked Immunosorbent Assay

Levels of C-X-C motif chemokine receptor 2 (CXCR2) and interleukin (IL)-8 in the plasma of patients with ILD-pSS and non-ILD-pSS were quantified using Human CXCR2 and Human IL-8 ELISA kits (Xitang Biology, Shanghai, China), respectively according to the manufacturer’s instructions. The color was developed using 3,3′,5,5′-tetramethylbenzidine (Sigma-Aldrich, St. Louis, MO, United States) and then measured using an ELISA plate reader (450 nm; Bio-Rad). All samples were measured in triplicate.

### Statistical Analysis

The data analysis was conducted using R version 3.6.1. Two-sample *t*-test was performed, calculating the mean ± standard error (SE). Differentially expressed genes (DEGs) were identified according to the following criteria: adjusted *p* value < 0.05 and absolute value of log2 fold change >1. Pearson’s correlation analysis was used to quantify the associations between gene-expression and clinical parameters, and Wilcoxon’s test was applied to comparisons between subgroups. *p* values <0.05 were considered significant.

## Results

### Patient Characteristics

The characteristics of the 36 patients with ILD-pSS and the 128 patients with non-ILD-pSS are detailed in Table 1. The mean ± SE age of patients with ILD-pSS was 60 ± 11.9 years, which was significantly higher than that of patients with non-ILD-pSS (49 ± 12.8 years). Both groups had similar proportions of men (19.4 and 14.1%, respectively) and higher proportions of women (80.6 and 85.9%, respectively).

**TABLE 1:** Clinical characteristics of patients with ILD-pSS and non-ILD-pSS.

Characteristic	ILD-pSS	Non-ILD-pSS	*p* value
*N*	36	128	
Age, years, mean (range)	60 (43–86)	49 (28–79)	0.000^**^
Male, *n* (%)	7 (19.4)	18 (14.1)	0.645
Anti-SSA antibodies, *n* (%)	23 (63.9)	97 (75.8)	0.097
Anti-SSB antibodies, *n* (%)	7 (19.5)	44 (34.3)	0.144
ESR (mm/h)	32.41 ± 18.52	23.34 ± 20.95	0.020^*^
CRP (mg/L)	10.26 ± 15.75	4.93 ± 9.30	0.059
IgG (g/L)	17.52 ± 4.44	18.20 ± 6.10	0.544
IgA (g/L)	3.28 ± 1.10	3.40 ± 1.43	0.639
IgM (g/L)	1.63 ± 1.30	1.35 ± 0.63	0.097
C3 (g/L)	1.09 ± 0.167	1.05 ± 0.22	0.320
C4 (g/L)	0.21 ± 0.17	0.22 ± 0.13	0.509
ESSDAI	20.64 ± 9.37	7.02 ± 7.85	0.000^**^

Data are means ± standard error, except where otherwise indicated.

*p* < 0.05, **p < 0.001 for ILD-pSS vs. non-ILD-pSS.

C3, complement 3; C4, complement 4; CRP, C-reactive protein; ESR, erythrocyte sedimentation rate; ESSDAI, EULAR Sjögren’s Syndrome disease Activity Index; Ig, immunoglobulin.

**FIGURE 1:**
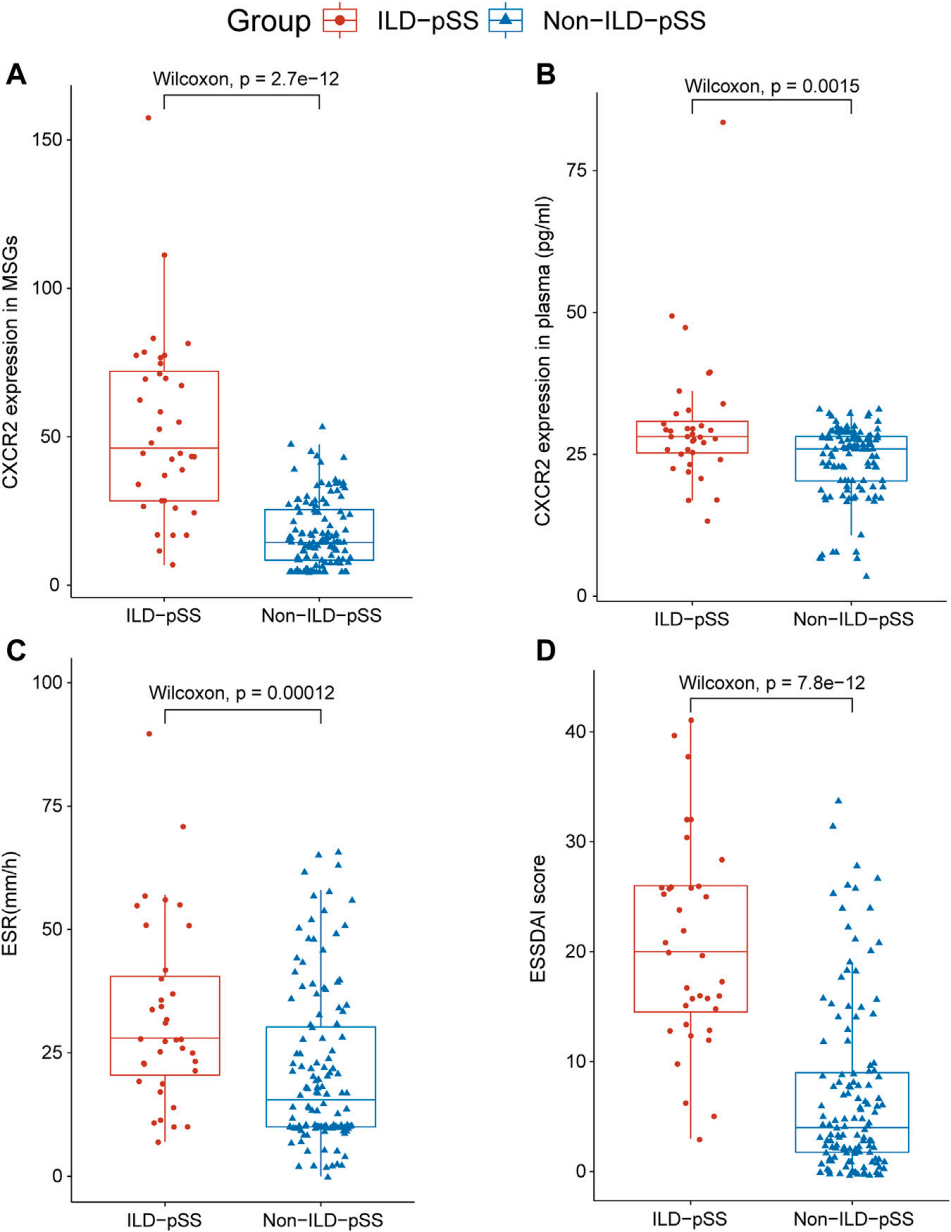
Analysis of *CXCR2* and IL-8 expression and clinical parameters in patients with ILD-pSS and non- ILD-pSS. *CXCR2* gene expression by RNA sequencing **(A)**, CXCR2 level in plasma **(B)**, ESR **(C)**, and ESSDAI scores **(D)** were significantly higher in patients with ILD-pSS vs. non-ILD-pSS patients. Horizontal lines, from bottom to top, denote the minimum, 25th percentile, median, 75th percentile, and maximum.

We also compared the differences in other clinical features between the two groups. Mean (±SE) ESSDAI scores were elevated in those with ILD-pSS (20.64 ± 9.37) and significantly higher than in patients with non-ILD-pSS (7.02 ± 7.85, *p* < 0.001; Figure 1D). Moreover, the mean (±SE) erythrocyte sedimentation rate (ESR) was higher in patients with ILD-pSS (32.41 ± 18.52 mm/h) vs. non-ILD-pSS (23.34 ± 20.95 mm/h, *p* < 0.001; Figure 1C). Likewise, mean (±SE) C-reactive protein levels were higher in ILD-pSS (10.26 ± 15.75 mg/L) than in non-ILD-pSS (4.93 ± 9.30 mg/L, *p* = 0.059; Table 1), although this did not reach statistical significance. There were no significant differences between the two groups in the presence of anti-SSA antibodies (*p* = 0.097) or anti-SSB antibodies (*p* = 0.144), or in the levels of immunoglobulin (Ig)G, IgA, IgM, C3, or C4 (*p* = 0.544, 0.639, 0.097, 0.320, and 0.509, respectively; Table 1).

### Identification of DEGs in Patients with pSS-ILD

**FIGURE 2:**
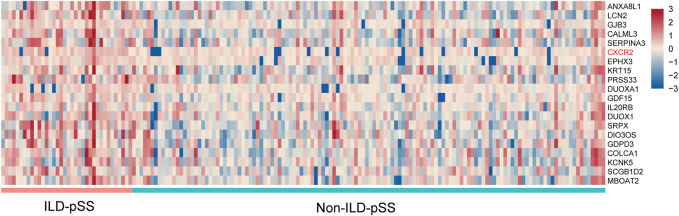
Heatmap of the top 20 DEGs between ILD-pSS and non-ILD-pSS.

We investigated the gene-expression profiles of MSGs from 36 patients with ILD-pSS and 128 with non-ILD-pSS using RNA sequencing. Several genes were found to be differentially expressed in ILD-pSS vs. non-ILD-pSS. The top 20 DEGs are shown in Figure 2.

### Clinical Correlations of Top 20 DEGs in ILD-pSS

**FIGURE 3:**
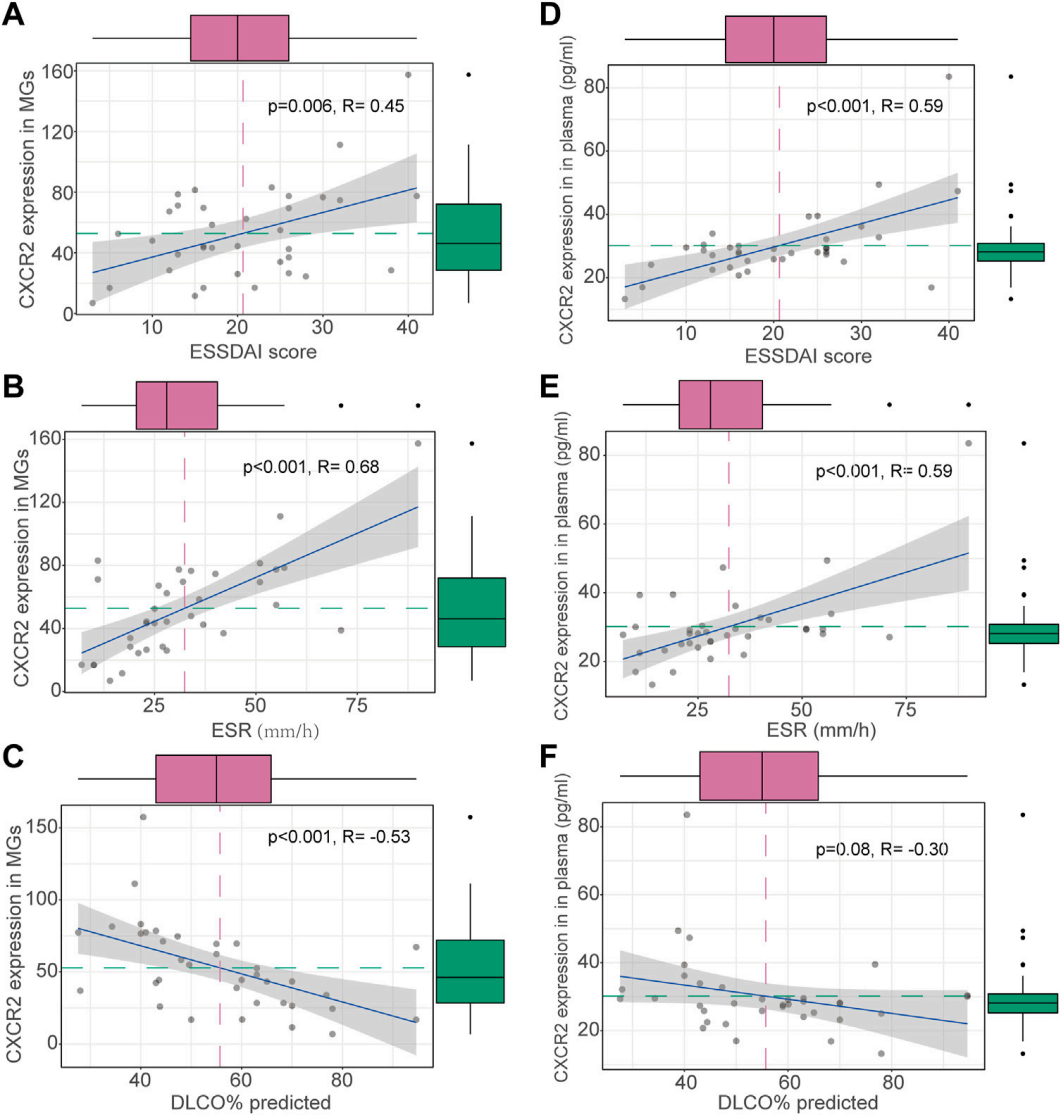
Clinical correlations of CXCR2 expression in patients with ILD-pSS. *CXCR2* gene expression in MSG was positively correlated with the ESSDAI score **(A)** and ESR **(B)**, and inversely associated with DLCO **(C)**. CXCR2 gene expression in plasma was similarly positively correlated with the ESSDAI score **(D)** and ESR **(E)**, and inversely associated with DLCO **(F)**.

To identify potential biomarkers for evaluating disease severity in patients with ILD-pSS, the clinical implications of the top 20 DEGs (Figure 2) were evaluated, with clinical parameters including ESSDAI score, ESR, C-reactive protein, forced vital capacity, forced expiratory volume in 1 s, and carbon monoxide diffusing capacity of the lung (DLCO). Among which *CXCR2*, a receptor for IL-8, had a higher relationship with clinical parameters than the other 19 genes (data not shown). In addition, *CXCR2* was of greatest interest because of its potential role in inflammation (Figure 1A) (Russo et al., 2009; Konrad and Reutershan, 2012; Liang et al., 2016; Hoegl et al., 2017). In our study, *CXCR2* was positively correlated with the ESSDAI score (*r* = 0.45, *p* = 0.006; Figure 3A) and ESR (*r* = 0.68, *p* < 0.001; Figure 3B). Moreover, *CXCR2* gene expression inversely associated with DLCO (*r* = −0.53, *p* < 0.001; Figure 3C). No correlations were found between *CXCR2* RNA expression by RNA sequencing and the other clinical parameters investigated.

### Circulating Chemokine Assay of CXCR2 and IL-8

Concentrations of CXCR2 and IL-8 in plasma were detected by ELISA in patients with ILD-pSS and non-ILD-pSS. CXCR2 levels were higher in those with ILD-pSS (*p* = 0.0015; Figure 1B), but there was no significant difference in plasma IL-8 levels between the two groups (*p* = 0.080). Concentrations of CXCR2 in plasma were positively correlated with the ESSDAI score (*r* = 0.59, *p* < 0.001; Figure 3D) and ESR (*r* = 0.59, *p* < 0.001; Figure 3E) in patients with ILD-pSS. Furthermore, CXCR2 levels were inversely associated with DLCO, although this did not reach statistical significance (*r* = –0.30, *p* = 0.08; Figure 3F).

## Discussion

In this study, we first compared the MSG gene-expression profiles of 36 patients with ILD-pSS and 128 patients with non-ILD-pSS, and identified several genes that are up-regulated in ILD-pSS. To identify potential biomarkers for evaluating disease severity in ILD-pSS, the clinical implications of the top 20 DEGs were examined. In particular, we found that patients with ILD-pSS had abnormally increased levels of *CXCR2*. Moreover, the *CXCR2* level correlated with clinical features in these patients, implying a potential role for *CXCR2* in the progression of ILD in pSS.

*CXCR2* (Rot and von Andrian, 2004) is a G-protein-coupled receptor assembled by seven transmembrane proteins that is activated by CXC chemokines containing the ELR (Glu-Leu-Arg) motif, including IL-8. *CXCR2* is expressed by granulocytes, especially neutrophils (Kim, 2004; Bertini et al., 2012). Neutrophils are a vital component of the innate immune system and play an essential role in the progression of lung fibrosis. In addition, research has shown that *CXCR2*-mediated neutrophil recruitment is essential for lung fibrosis (Russo et al., 2009; Konrad and Reutershan, 2012; Liang et al., 2016; Hoegl et al., 2017), and that blocking *CXCR2* can reduce lung fibrosis (Russo et al., 2009). In this study, *CXCR2* was found to be elevated in both the MSG and plasma of patients with ILD-pSS vs. non-ILD-pSS, with the results showing that *CXCR2* may also play an essential part in organ-typical damage and circular inflammation in pSS. Although patients with ILD-pSS and those with non-ILD-pSS may initially show similar clinical manifestations (Nannini et al., 2013), those with ILD-pSS can have a poorer prognosis (Roca et al., 2017; Sambataro et al., 2020). Further molecular functional investigation of CXCR2 in ILD-pSS progression may have significant implications.

The ESR is a common inflammatory marker, and an elevated ESR is found associated with ILD underlying other connective tissue diseases (Young et al., 2007; Jung et al., 2018). Furthermore, previous research has demonstrated that the ESR is higher in patients with ILD-pSS vs. non-ILD-pSS (Li et al., 2015; Dong et al., 2018). Our study confirmed this, with a higher ESR in patients with ILD-pSS. Moreover, *CXCR2* in both MSG and plasma was highly associated with the ESR, indicating that *CXCR2* may help differentiate ILD-pSS from non-ILD-pSS in some situations. The ESSDAI was developed to measure systemic disease activity in pSS (Seror et al., 2010; Risselada et al., 2012). In our study, ESSDAI scores were much higher in patients with ILD-pSS vs. non-ILD-pSS. Furthermore, ESSDAI scores were highly associated with *CXCR2* levels in both MSG and plasma from patients with ILD-pSS, indicating that *CXCR2* may serve as a useful candidate for evaluating disease severity. DLCO is one measure for predicting lung function. Most previous studies (Chen et al., 2016; Barnes et al., 2018) used a significant decline in DLCO to define ILD worsening. In our study, *CXCR2* levels in both MSG and plasma from patients with ILD-pSS were inversely associated with DLCO, confirming the role of *CXCR2* in evaluating the disease severity of ILD in those with pSS.

To our knowledge, this is the first study to suggest a possible role for *CXCR2* in ILD-pSS. We observed extensive expression of *CXCR2* in both MSG and plasma in patients with ILD-pSS, and many manifestations of ILD are characterized by the accumulation of inflammatory cells. *CXCR2* is implicated in neutrophil infiltration and can be activated by many other CXC chemokines in inflammatory process. Therefore, the contribution of *CXCR2* to the inflammatory and further fibrotic process in ILD-pSS should be further investigated.

## Conclusion

Our results provide new insights into the role of *CXCR2* in patients with ILD-pSS. *CXCR2* may serve as a potential biomarker for evaluating disease severity in this population.

## Data Availability

The datasets presented in this study can be found in online repositories. The names of the repository/repositories and accession number(s) can be found below: Gene Expression Omnibus, GSE171896.
